# Weight-Gain in Psychiatric Treatment: Risks, Implications, and Strategies for Prevention and Management

**DOI:** 10.4103/0973-1229.58819

**Published:** 2010

**Authors:** Amresh Shrivastava, Megan E. Johnston

**Affiliations:** * *The University of Western Ontario, Department of Psychiatry, & Associate Scientist, Lawson health Research Institute, London, ON, Canada*; ** *University of Toronto, Department of Psychology, Toronto, ON, Canada*

**Keywords:** *Weight gain*, *psychiatric patients*, *antipsychotics*, *antidepressants*, *treatment-induced weight-gain*, *psychopharmacology*

## Abstract

Weight-gain in psychiatric populations is a common clinical challenge. Many patients suffering from mental disorders, when exposed to psychotropic medications, gain significant weight with or without other side-effects. In addition to reducing the patients’ willingness to comply with treatment, this weight-gain may create added psychological or physiological problems that need to be addressed. Thus, it is critical that clinicians take precautions to monitor and control weight-gain and take into account and treat all problems facing an individual. In this review, we examine some of the key issues surrounding weight-gain in individuals suffering from mental disorders for contemporary practitioners in community clinics. We describe some factors known to make certain patients more susceptible to treatment-induced weight-gain and mechanisms implicated in this process. We also highlight a few psychological and pharmacological interventions that have proven effective in weight management. Importantly, we provide critical steps for management and prevention of weight-gain and related issues in the clinical practice of psychopharmacology.

## Introduction

Weight-gain in psychiatric populations is a common clinical challenge. Many patients suffering from mental disorders, when exposed to psychotropic medications, gain significant weight with or without other side effects. Being overweight or obese has been acknowledged as a public health problem due to its correlation with mortality and increased comorbidity of other physical disorders. This association requires new paradigms of management of psychiatric disorders that take into account comorbid physical disorders.

When treated over a short period of time, weight-gain may be minimal and reversible once a drug is discontinued. With long-term treatment, however, psychiatric patients may gain a significant amount of weight, possibly reducing their willingness to comply with treatment. Additionally, this weight-gain may create added psychological or physiological problems that need to be addressed. Thus, it is critical that clinicians take precautions to monitor and control weight-gain to take into account and treat all problems facing such an individual.

In this review, we examine some of the key issues surrounding weight-gain in individuals suffering from mental disorders for contemporary practitioners in community clinics. We discuss measures that can be adopted in practice to deal with this issue while optimizing treatment and outcome. We start by providing an overview for practicing clinicians on the evidence and course of weight-gain during psychiatric treatment and some of the issues this entails. We describe some factors known to make certain patients more susceptible to treatment-induced weight-gain and mechanisms implicated in this process. Finally, we provide critical steps for management and prevention of weight-gain and related issues in the clinical practice of psychopharmacology.

## Evidence of Weight-gain

The prevalence of obesity is increasing at an alarming rate. This has led to an increase in research into the causes, comorbidities, and treatment of obesity in recent years. Clinical studies indicate that a high prevalence of metabolic syndrome exists in individuals afflicted with serious mental illnesses, particularly those with schizophrenia. In addition, psychotropic agents, including antipsychotic medications and antidepressants, have been found to be associated with substantial weight-gain (Newcomer, 2007). This weight-gain is troublesome as it increases an individual’s risk of diabetes and cardiovascular disease. A normal body mass index (BMI) is considered to be between 18.5 and 24.9, a BMI between 25 and 29.9 is classified as overweight, and 30 to 39.9 denotes obesity. Patients with a BMI above 40 are considered extremely obese (Morrato, 2009)

Research examining the differential effects of various antipsychotic medications has shown that both the frequency as well as the amount of weight-gained is high in patients treated with olanzapine (average gain of 2.3 kg/month), clozapine (1.7 kg/month), quetiapine (1.8 kg/month), and zotepine (2.3 kg/month), (Wetterling, 2001). Additionally, they also report that some changes in weight have also been observed in treatment with risperidone (average gain of 1.0 kg/month), and ziprasidone seems to induce only small changes in weight (0.8 kg/month). Overall, the largest body of research exists to support an association between weight-gain and treatment with olazapine and clozapine (Gebhardt *et al*. 2009; Haddad, 2005).

The strength of the causal relationship between antipsychotic drug exposure and weight-gain can be assessed using a drugs trial conducted with antipsychotic-naive patients. Tarricone and colleagues (2009) reviewed 11 studies reporting the effects of antipsychotic drugs on body weight in patients naïve to antipsychotic drugs. The mean values of weight-gain in these patients were highly significant from the first few weeks of treatment. The sample averaged around 3.8 kg in gained weight and an increase of 1.2 in body mass index (BMI). Thus, weight-gain associated with antipsychotic drug treatment appears to occur rapidly in the first few weeks and continue during the following months (Tarricone *et al*.. 2009).

Weight-gain is not restricted to individuals treated with antipsychotics; antidepressants and lithium have also been shown to lead to unwanted weight-gain. Studies have found that antidepressants lead to an increase of weight in anywhere between 24-100% of patients, with an average weight-gain of 0.57 to 1.37 kg per month of treatment (Fava, 2000; Garland *et al*. 1988). Lithium carbonate therapy is also associated with significant weight-gain, with some studies reporting a gain of over 10 kg in 20% of patients (Livingstone& Rampes, 2006; Vestergaard *et al*. 1980).

It should be noted that not all psychotropic drugs lead to weight-gain, and some have even been shown to decrease weight, such as serotonin-reuptake inhibitors (SSRI) during the first few weeks of use (Michelson *et al*. 2000), felbamate (Bergen *et al*. 1995), and topiramate (Dursun& Devarajan, 2000).

### When does weight-gain occur?

In their sample of bipolar patients, Fagiolini *et al*. (2002) found that most weight-gain occurred during acute treatment rather than during maintenance treatment. This research demonstrated the benefit of maintenance treatment as minimal weight was gained during the maintenance phase, whereas acute depressive episodes were related to weight-gain. Also, stabilization on maintenance medication facilitates participation in interventions directed specifically at weight loss (Fagiolini *et al*. 2002).

In patients treated with clozapine, Umbricht *et al*. (1994) found that significant weight-gain occurred primarily during the first six to 12 months, and continued into the third year of treatment. These researchers found that being underweight at baseline was correlated with a greater amount of weight-gained, while overweight status at baseline was associated with a higher final weight following treatment than those who were not overweight at baseline.

Several long-term naturalistic studies found that weight-gain is less marked in the long term than in controlled trials of a shorter or comparable duration. With the use of many antipsychotics, weight may stabilize in the short to medium term but it appears that weight-gain continues beyond the first year when treated with clozapine (Haddad, 2005). Some predictors of long-term weight-gain include a lower body mass index, a rapid initial increase in weight, and increased appetite. Weight-gain also seems to be greater in first onset patients due to their lack of prior antipsychotic treatment and the weight-gain associated with these treatments (Haddad, 2005). Fortunately, it does seem that weight-gain resulting from antipsychotics occurs primarily during the first two years of treatment and then levels off (Silverstone *et al*. 1988, Allison, 2009).

### Is Weight-gain dose-dependent?

A recent review attempted to answer the question of whether weight-gain and associated metabolic changes are dose-dependent (Simon *et al*. 2009). A relationship appears to exist between the administered dose of clozapine and olanzapine and metabolic outcomes. With regard to risperidone and other antipsychotic medications, further research is required to make an accurate assessment of a possible dose-dependency for weight-gain (Simon *et al*. 2009). However, the relationship between clozapine and olanzapine plasma concentrations and metabolic disturbances provide evidence for a causal effect of antipsychotic medications on weight-gain.

## Clinical Impact of Weight-gain

### Morbidity, mortality, and physical health

Research suggests that individuals with severe mental illness have significantly worse health outcomes and premature mortality than the general population. Individuals with schizophrenia have up to a 20% shorter lifespan compared to the general population, with cardiovascular disease representing the most common cause of death (Newcomer, 2007). Many factors are implicated in the poor health of individuals with schizophrenia, including increased prevalence of smoking, poverty, and poor nutrition (Newcomer, 2007); additional contributions are made by the adverse metabolic side effects of antipsychotic medications, including weight-gain (Amiel *et al*. 2008). An important aspect of managing mental illness is managing the side effects of antipsychotics using a combination of administrative, behavioral and medical approaches (Amiel *et al*. 2008).

An additional issue is that overweight and obese individuals are at risk for numerous psychological and physiological health problems, such as depression and disordered eating (Bean *et al*. 2008). Hence, mental health professionals need to take special care in the case of patients with obesity, to watch for and treat these additional health concerns if they should arise. Evidence suggests that mentally ill patients often do not receive adequate care for their medical illnesses, highlighting the need for increased awareness of and attention to the physical health problems of individuals with mental illness (Newcomer, 2007). In particular, the metabolic and weight issues resulting from antipsychotic treatments require appropriate management.

## Weight-gain in Specific Conditions

### Affective disorders

Major depressive disorder can be a chronic condition involving recurrent episodes throughout a patient’s life. In order to reduce the chance of relapse, long-term treatment with antidepressants is necessary. Unfortunately, many patients choose to discontinue medication due to long-term side effects resulting from these drugs, one of which is weight-gain (Moller, 2008). In one group of individuals with bipolar disorder, Fagiolini and colleagues (2002) found that 68% of the patients were obese or overweight at entry into the study; 32% of the individuals in the study were classified as obese. Additionally, it was found that the number of previous depressive episodes experienced by an individual was associated with being overweight or obese at study entry (Fagiolini *et al*. 2002). Thus, weight-gain seems especially prevalent in affective disorders, although this likely results from both the effects of the illness as well as treatment effects. Clearly, in this group of patients, weight management and control is particularly critical to include as part of a treatment program.

### Childhood and adolescence

The prevalence of pediatric obesity is rising in both developed and developing countries. As overweight children and adolescents are at an increased risk of medical comorbidities and psychosocial and behavioral difficulties, this makes antipsychotic-induced weight-gain a significant public health concern (Jelalian *et al*. 2007). Children and adolescents are known to be at a higher risk for weight-gain associated with antipsychotic treatment (Citrome& Vreeland, 2009). A recent study looked at antipsychotic-induced weight-gain in a pediatric sample and noted marked and rapid weight-gain (Correll *et al*. 2009). Children and adolescents between the ages of 4 and 18 were treated with aripiprazole, olanzapine, quetiapine, or risperidone for 12 weeks and results showed an average weight-gain between 4.4 and 8.5 kg depending on the agent (highest gain was in olanzapine patients, lowest gain in aripiprazole patients).

Many current pediatric weight control interventions proven to be effective in research trials are limited by samples that may exclude participants with psychiatric co-morbidities (Jelalian *et al*. 2007). Thus, it is important that clinicians treating overweight and obese children and adolescents with psychiatric disorder assess individual, familial, and contextual variables specific to weight in order to prioritize treatment objectives. Similar to adults, weight-gain is an important consideration for practitioners treating children and adolescents with antipsychotics especially, as the detrimental effects of weight-gain, both psychological and physiological, may manifest to a greater degree in children. Future research is needed to explore these issues.

### Pregnancy

Many women with psychotic disorders have children at some point in their lives, leading to a new set of issues. Women with schizophrenia receive less prenatal care and have poorer health, resulting in many health risks for their infants (Howard, 2005). McKenna *et al*. (2005) followed pregnant women taking atypical antipsychotics (olanzapine, risperidone, quetiapine, and clozapine) and found a greater BMI in the mothers and lower birth weight in the infants. Weight-gain and increased BMI pose many health risks for pregnant women as obesity is associated with obstetric complications, including gestational diabetes mellitus, pre-eclampsia, and caesarean delivery (Brost *et al*. 1997). Obesity also poses a risk to the children they are carrying. Boney *et al*. (2005) found that children exposed to maternal obesity in the womb were more likely to have metabolic syndrome themselves, and pregnancies in obese women are more likely to result in stillbirth and neonatal deaths than pregnancies in women of normal weight (Kristensen, 2005). Thus, weight-gain as a result of antipsychotic medication can pose additional risks to women who are pregnant and may result in negative health consequences for these mothers and their infants.

### Dementia

Body mass index (BMI) may influence or be influenced by the brain structures and functions involved in dementia processes (Gustafson, 2008). The adipose tissue associated with BMI changes over the lifespan and is related to brain development in terms of cognitive functioning, intelligence, and cognitive disorders such as dementia. In general, lower BMIs and correspondingly greater rates of weight decline during the years preceding dementia onset, are related to dementia. Risk of dementia is increased, however, by a high BMI during mid-life or in the 5-10 years preceding dementia onset (Gustafson, 2008)

## Public Health

The weight-gain associated with antipsychotic medications represents a liability to the public health system. A variety of factors make schizophrenia an economic burden on society, including unemployment, incarceration, and healthcare (Goeree *et al*. 2005); but obesity represents an additional factor adding to this burden. It may be more difficult to treat obesity in individuals who have gained weight as a result of antipsychotic treatment as their medication increases appetite and produces fatigue and the illness itself decreases motivation and social activities (Centorrino *et al*. 2006). Thus, these individuals who have gained weight as a result of their psychiatric treatment are an additional cost to the healthcare system. The medical and health risks associated with obesity result in a cost to society beyond that of psychiatric care alone.

## Individual Susceptibility

Research using data from twin, adoption, and family studies suggests that at least 50% of individual difference in body mass index (BMI) is due to genetic factors. However, the increase in obesity rates over recent years illustrates the impact of environmental factors on body weight (Hebebrand& Hinney, 2008). Males and females are also differentially susceptible to weight-gain. Gender differences are apparent in how and where body fat is stored, as men amass more fat in the intra-abdominal area than pre-menopausal women. This increases males’ risk of developing cardiovascular problems, type-2 diabetes mellitus, certain cancers and other metabolic problems that relate to obesity (Shi& Clegg, 2009).

The increased appetite, and associated weight-gain, resulting from cannabis use has been documented. Most studies have focused on short-term outcomes, however, and the long-term effects of cannabis use are unclear (Mushtaq *et al*. 2008). A review by Mushtaq and colleagues (2008) suggests that cannabis use in patients with psychosis may be associated with increased body weight, and these authors concluded that cannabis use may be one factor contributing to the weight and health-related problems of this patient group.

### Predictors of antipsychotic-induced weight-gain

Research indicates that antipsychotic-induced weight-gain is predicted by higher parental BMI, patients’ premorbid BMI, the female gender, younger age, and non-smoking status (Gebhardt *et al*. 2009). These findings suggest that there is a strong impact of predispositional factors on weight-gain, beyond treatment factors. Additionally, Gebhardt *et al*. (2009) found that the diagnosis of a schizophrenia spectrum disorder was related to an increased BMI and suggest that this may result from a longer duration of atypical antipsychotic treatment. Similarly, Saddichha *et al*. (2008) examined a group of patients diagnosed with first-episode schizophrenia and found that waist circumference and weight at baseline, as well as antipsychotic use, were related to greater weight-gain. When looking at the impact of different medications on weight-gain, olanzapine lead to greater weight-gain as compared to risperidone and haloperidol (Saddichha *et al*. 2008).

## Biological Mechanisms of Weight-gain

The underlying pathomechanism behind weight-gain in response to antipsychotic treatment remains, for the most part, unclear. The strongest correlate of gains in body weight discovered so far is the relative receptor affinities of the atypical antipsychotics for histamine H1 receptors; also important is the ratio of their affinity for serotonin 5-HT2 and dopamine D2 receptors (Wetterling, 2001).

In the past, some of the adverse effects of atypical antipsychotic treatment have been associated with the antagonism of monoamine receptors; more recent data, however, indicate that metabolic effects (e.g. hypertriglyceridemia, impaired glucose/insulin homeostasis) may not be related to these mechanisms (Houseknecht *et al*. 2007). New theories of the mechanisms underlying antipsychotic-associated weight-gain focus on the effect of antipsychotics on peptide hormonal regulators of metabolic control, including leptin, ghrelin, and adiponectin. Jin and colleagues (2008) found that the weight-gain associated with medication was directly related to changes in leptin; there were no added antipsychotic effects on leptin signaling. However, long-term studies on ghrelin showed increased levels in patients on atypical antipsychotics that typically produce weight-gain. Thus, it appears that ghrelin, and possibly other peptide hormones, may be useful predictors of weight-gain in patients who are receiving antipsychotic treatments (Jin *et al*. 2008).

Tricyclic antidepressants have been shown to increase appetite and carbohydrate cravings (Garland *et al*., 1988). Additionally, decreased energy expenditure may contribute to weight-gain (Fernstrom *et al*. 1985; Korner& Aronne 2003). In the case of lithium carbonate therapy, research has shown an insulin-like effect on carbohydrate metabolism, altered fat cell metabolism, and depressed thyroid function (Ackerman& Nolan 1998; Garland *et al*. 1988).

**Table 1:** Efficacy of weight control with medication

Medication	Efficacy	Study
Ephedrine	Body weight reduction of 24kg in 24 weeks	Astrup *et al*. 1991
Sibutramine	Loss of 5% of initial weight in 24 weeks	Apfelbaum *et al*. 1999
Orlistat	Loss of 5% of initial weight in 24 weeks	Finer *et al*. 2000
Topiramate	Weight reducing effect in combination with clozapine	Dursun & Devarajan, 2000
Metformin	Helpful in reversing weight-gain in pediatric patients	Morrison *et al*. 2002
Naltrexone	Reduction in food craving; reversal or control of weight-gain	Zimmermann *et al*. 1997
Amantadine	Weight loss of 3.5kg in 3-6 months	Floris *et al*. 2001

## Psychological and Pharmacological Interventions

There are various pharmacological (e.g. switching medications) and nonpharmalogical (e.g. diet and exercise) interventions for patients who have gained weight as a result of psychiatric treatment. It seems that modest short-term weight loss is possible with either type of intervention. The drug reboxetine (4mg daily for 6 weeks) appears effective for weight prevention while topiramate (100-200mg daily for 12 weeks) is useful for both prevention and for established weight-gain (Faulkner& Cohn 2006; Faulkner *et al*. 2007). Additional research has shown topiramate to result in substantial weight loss when combined with valproate or clozapine (Gordon& Price 1999; Dursun& Devarajan 2000, Afshar 2009).

Sibutramine, an SSRI licensed for weight loss, has demonstrated significant weight reduction in several double-blind placebo-controlled trials (Apfelbaum *et al*. 1999, Payer *et al*. 2004). In these studies, patients lost about 5% of their initial weight and maintained this for at least one year, primarily due to reduced appetite and an increase in energy expenditure (Apfelbaum *et al*. 1999,). Similar weight loss effects to sibutramine are seen with orlistat, which reduces intestinal absorption of fat (Finer *et al*. 2000). Additionally, metformin, an anti-diabetic drug, can help reduce the weight-gained in response to olanzapine, valproate, and risperidone (Morrison *et al*. 2002, Chen *et al*. 2008, Arman, 2008).

**Figure 1:**
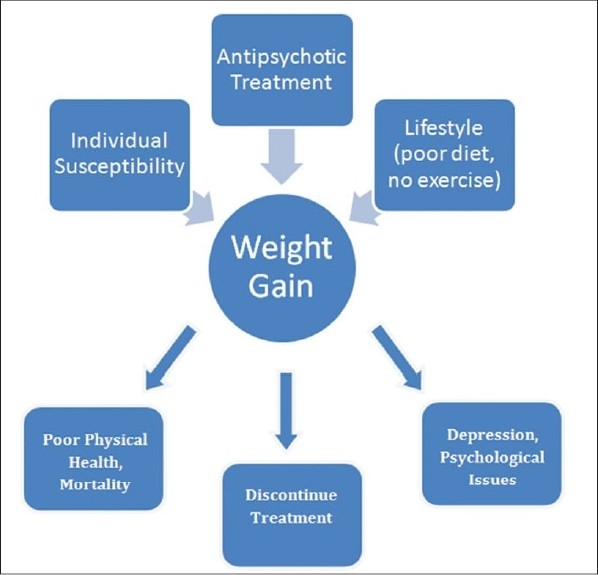
Causes and effects of weight-gain in psychiatric treatment

Modifications to diet and physical activity can also be effective. In particular, cognitive/behavioral interventions that provide strategies for adhering to diet and exercise lifestyle modifications have proven to be valuable for weight management (Weber& Wyne, 2006). They implemented a cognitive/behavioral group intervention modeled after the Diabetes Prevention Project in a group of patients diagnosed with schizophrenia or schizoaffective taking atypical antipsychotics. After 16 weeks it was found that the intervention patients lost more weight (2.9% of body weight) than a control “treatment as usual” group (0.6% body weight). This intervention group consisted of weekly sessions which centered on various strategies, such as goal setting, discussions on barriers to change, and plans to increase physical activity. Participants in the intervention also had to keep a food and activity journal, which was submitted at each weekly session (Weber& Wyne, 2006).

Thus, there are interventions strategies available to prevent weight-gain and its associated health risks in individuals undergoing psychiatric treatment. Both pharmacological and nonpharmacological strategies show promise in weight reduction. Overall it seems that the best approach is to use pharmacological interventions in conjunction with dietary and behavioral modifications (Faulkner, 2007). However, given the modest effect of these interventions, appraising metabolic risk is a critical first step to preventing weight-gain in patients starting on antipsychotics or antidepressants. Additionally, in extreme cases, surgery remains an option when other weight control methods have failed and obesity-related co-morbidities and mortality become a concern (Expert panel on the identification, 1998). Further research is required to determine which methods of intervention show the best long-term effects and what individual differences influence the type of intervention that will be effective.

## Managing Weight-gain in Clinical Practice: Management and Prevention

The weight-gain that can result from treatment with antipsychotic medication may lead some individuals to discontinue medication, inhibiting their potential for improved mental health (Monteleone *et al*. 2009). For those who do continue with their medication, the associated weight-gain can lead to numerous other health and psychosocial problems. Citrome (Bhuvaneshwar *et al*. 2009) and Vreeland (2009) report that by monitoring body weight early in treatment, practitioners would be able to better predict patients who are at high risk for substantial weight-gain. In this way, excessive weight-gain can be prevented before it becomes an impediment to the improvement of mental health.

Switching antipsychotic medication is one method to reduce body weight, although this may not be clinically feasible. Switching from one drug to another is a clinical decision depending on several factors e.g. tolerance, safety and efficacy of molecules used. Such decisions are always to be taken in the best interest of the patient depending on the existent state of knowledge.

Evidence for the effectiveness of adjunctive medication strategies is conflicting; however, lifestyle therapies and other non-pharmacological interventions have proven successful in controlled clinical trials (Citrome& Vreeland, 2009). Life style treatment includes cognitive behavioral and educational psychotherapy, regular physical fitness programs, preferably supervised, follow-up of dietary regimes, and traditionally accepted long walks. All these are clubbed under the rubric of non-pharmacologic interventions.

Kerwin (2009) reports on a panel of European experts in the field of schizophrenia who met to discuss improved treatment monitoring as a means of optimizing patient management. The panel agreed that weight-gain was one of the core parameters to be monitored in all patients with schizophrenia and that optimizing treatment requires an individualized management strategy. Kerwin (2009) highlights the fact that treatment strategies for individuals with schizophrenia need to be switched from medication-based to more holistic approaches. This would include a multidisciplinary team that would be able to address the physical health problems experienced by many individuals with schizophrenia. Psychiatric and general health care needs to be integrated as much as possible to optimize outcomes (Wadden *et al*. 2007). In addition to continued patient-practitioner contact, long-term use of pharmacotherapy combined with lifestyle modification (diet, physical activity, and behavioral therapy) appears important for long-term weight control (Wadden *et al*. 2007). Three medications for weight loss and maintenance, sibutramine, orlistat, and rimonabant have proven to result in a weight loss of 7-10% of initial body weight in one year of treatment (Bray, 2007). By maintaining communication with primary care physicians and monitoring for weight-gain psychiatrists can help to maintain the physical health of patients.

## Best Way Forward

The best way forward in management and prevention is to be vigilant from the very beginning. Specific measures are required in the clinical practice of psychopharmacology to deal with weight-gain and related issues:

1. Thorough baseline assessment of family history, risk factors, health psychology, life style and dietary habits.
2. Monitor weight and metabolic parameters closely throughout the course of treatment.
3. Work with a meaningful multidisciplinary team to targ*et al*l vulnerable areas.
4. Incorporate behavioral intervention programs.
5. Involve dieticians to monitor nutritional requirement.
6. Avoid polypharmacy as much as possible.
7. Attempt to treat weight-gain with behavioral and pharmacological measures.
8. Treat metabolic conditions, like hyperlipidemia and diabetes.
9. Obtain good control over hypertension.
10. Obtain adequate remission of depressive and negative symptoms.
11. Implement motivational therapy when required.
12. Equip clinics with all necessary resources under one umbrella for feasibility.

## Summary and Conclusion

Many patients suffering from mental disorders, when exposed to psychotropic medications, gain a significant amount of weight; a trend acknowledged as a public health problem due to its correlation with mortality and increased comorbidity of other physical disorders. This association requires new paradigms of management of psychiatric disorders that take into account co-morbid physical disorders. An important aspect of managing side effects of antipsychotics and antidepressants is to use a combination of administrative, behavioral and medical approaches to assess and treat all problems that an individual faces. Obesity represents a burden both to the individual and to society and requires appropriate attention. If feasible, switching medication may be one solution. In many cases, weight loss (or weight control) programs will need to be incorporated into an individual holistic treatment plan.

### Take home message

In sum, medication-induced weight-gain can be detrimental to a patient’s physical health and recovery process. To address this issue, a holistic, multidisciplinary approach to treatment is recommended. It is critical that clinicians take precautions to monitor and control weight-gain and to treat all problems facing a patient; the best way forward in management and prevention is to be vigilant from the very beginning.

### Conflict of interest

None declared

### Author contributions

The first author was responsible for the literature review and the second author was responsible for writing the article.

### Declaration

This manuscript is an original, unpublished piece. It has not been submitted for publication elsewhere.

## Questions that this Paper Raises

1. What are the mechanisms through which psychiatric medications cause weight-gain? Can this information be used to prevent treatment-induced weight-gain?
2. Which pharmacological and nonpharmacological interventions for patients who have gained weight produce the most weight loss and, more importantly, the best maintenance of weight lost over the long term?
3. Which cognitive-behavioral intervention strategies (e.g. goal setting, food journals) are most effective and feasible for individuals diagnosed with schizophrenia, affective disorders?
4. What are some practical and cost-effective strategies to incorporating a holistic, multidisciplinary approach to the management of every individual treated with psychiatric medications?
5. What are the effects of combining weight loss drugs with antipsychotic medications?

## About the Author


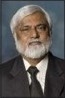
 *Amresh Shrivastava, MD, DPM, MRCPsych, is currently an Assistant Professor of Psychiatry at the University of Western Ontario in London, Ontario, Canada and an Associate Scientist at the Lawson Health Research Institute. His clinical work involves early psychosis and acute psychiatric work, and he is the Physician Team Leader for the Elgin Prevention and Early Psychosis Program (PEPP), Regional Mental Health Care – St. Thomas, Ontario, Canada. Dr. Shrivstava has also been the Executive Director of PRERANA Charitable Trust in Mumbai, India since 1992.*


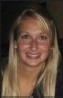
 *Megan Johnston, MA, is currently a PhD Candidate in Psychology at the University of Toronto in Toronto, Ontario, Canada. Her primary research interests involve parental and socialization influences on moral development and antisocial and pro-social behavior in adolescence. She is also affiliated with Regional Mental Health Care – St. Thomas, Ontario, Canada where her research focuses on the social and clinical outcomes of schizophrenia and suicide risk assessment and prevention.*
